# Naturally occurring variation in tadpole morphology and performance linked to predator regime

**DOI:** 10.1002/ece3.1538

**Published:** 2015-07-07

**Authors:** James B Johnson, Daniel Saenz, Cory K Adams, Toby J Hibbitts

**Affiliations:** 1 Department of Biology, Texas A&M UniversityCollege Station, Texas, 77843; 2 USDA Forest Service, Southern Research StationNacogdoches, Texas, 75965; 3 Biodiversity Research and Teaching Collections, Department of Wildlife and Fisheries Sciences, Texas A&M UniversityCollege Station, Texas, 77843

**Keywords:** Anuran larvae, fast-start performance, morphology, phenotypic integration, predation, tadpole

## Abstract

Divergent natural selection drives a considerable amount of the phenotypic and genetic variation observed in natural populations. For example, variation in the predator community can generate conflicting selection on behavioral, life-history, morphological, and performance traits. Differences in predator regime can subsequently increase phenotypic and genetic variations in the population and result in the evolution of reproductive barriers (ecological speciation) or phenotypic plasticity. We evaluated morphology and swimming performance in field collected Bronze Frog larvae (*Lithobates clamitans*) in ponds dominated by predatory fish and those dominated by invertebrate predators. Based on previous experimental findings, we hypothesized that tadpoles from fish-dominated ponds would have small bodies, long tails, and large tail muscles and that these features would facilitate fast-start speed. We also expected to see increased tail fin depth (i.e., the tail-lure morphology) in tadpoles from invertebrate-dominated ponds. Our results support our expectations with respect to morphology in affecting swimming performance of tadpoles in fish-dominated ponds. Furthermore, it is likely that divergent natural selection is playing a role in the diversification on morphology and locomotor performance in this system.

## Introduction

Integral to the adaptive process is that the phenotype directly determines an individual’s performance (e.g., fighting ability, locomotor performance, resource acquisition) and it is performance which directly determines fitness (Arnold 1983; Benkman 2003). Yet, species often encounter a diversity of conflicting environmental conditions that place a variety of demands on individuals throughout the species’ range, thus giving rise to divergent natural selection (Schluter 2001; Kawecki and Ebert 2004). Environmental heterogeneity selects for different aspects of performance which are facilitated by likewise divergent phenotypic traits (Losos 1990; Langerhans 2009b). Thus, divergent natural selection is a major mechanism in the production of diversity in biological systems and may lead to ecological speciation (Nosil & Crespi 2006; Schluter 2009) as well as the evolution of phenotypic plasticity (Van Buskirk 2002; DeWitt and Scheiner 2004).

A particularly powerful source of divergent selection is variation in local predator assemblage (Van Buskirk 2002; Nosil and Crespi 2006; Langerhans 2009a). Selection favors individuals displaying trait variation (e.g., morphology) which improves antipredator performance (e.g., escape performance, Langerhans 2009a; and detection avoidance Endler 1988), but the combination of antipredator performance and phenotypic traits which are successful is dependent on type and density of predators present in the local environment (Langerhans 2009b; Pruitt and Husak 2010). For example, the distribution of anuran larvae and their predators is strongly influenced by the frequency of drying events in local habitats (i.e., hydroperiod; Wellborn et al. 1996; Babbitt et al. 2003). Habitats which dry quickly have few if any predators (Babbitt et al. 2003). Intermediate sites that persist for a few to several months are often dominated by invertebrate predators (Babbitt et al. 2003) which are efficient predators of tadpoles (Van Buskirk and McCollum 2000a; Johnson et al. 2008). Conversely, permanent habitats may have predatory fish (Wellborn et al. 1996; Babbitt et al. 2003) which may greatly impact and even completely exclude some anuran species from these habitats (Bronmark and Edenhamn 1994; Wellborn et al. 1996; Tiberti and von Hardenberg 2012). As expected, fish and invertebrate predators have been shown to place very divergent demands on the larval anurans that occupy these habitats, particularly with respect to morphology and performance (Dayton et al. 2005; Teplitsky et al. 2005; Wilson et al. 2005; Touchon and Warkentin 2008).

In fish, fast-start swimming performance (i.e., high speed, rapid acceleration, and turning) can improve survival in the presence of larger piscivorous fish (Webb 1986; Walker et al. 2005; Langerhans 2009a). Some evidence suggests that in anuran larvae, fast-start swimming performance may also increase survival in the presence of fish predators as well (Teplitsky et al. 2005). Body shape plays a strong role in facilitating these evasive locomotor tactics. Animals with a large muscular propulsor relative to a small anterior region displaying increase fast-start performance (anuran literature reviewed by Arendt 2010; fish literature reviewed by Langerhans and Reznick 2010). Several studies have shown that in some anurans, these features are inducible when reared in the presence of large predatory fish (Teplitsky et al. 2003, 2005; Benard 2006; Touchon and Warkentin 2008; El Balaa and Blouin-Demers 2013) and that these induced features increase fast-start swimming performance (Teplitsky et al. 2005; El Balaa and Blouin-Demers 2013). This suggests, albeit indirectly, that fast-start swimming performance facilitated by large tail muscles and small bodies improves tadpole survival in the presence of fish predators.

Yet, increased locomotor performance is not the only morphological defense utilized by anuran larvae. In the presence of some invertebrate predators, such as dragonfly larva (aeshnids in particular), many species of tadpole develop a medially deep tail fin, which in some species are contrastingly colored (Van Buskirk et al. 1997; Relyea and Werner 2000). This induced medial tail fin depth does not increase fast-start performance (Van Buskirk and McCollum 2000b), and fast-start performance is of little importance to tadpole survival in the presence of larval Aeshnid predators (Johnson et al. 2008). Instead, this predator-induced medially deep tail directs attacks away from the more vulnerable body (Van Buskirk et al. 2003). Tail damage is common in habitats with an abundance of invertebrate predators (Blair and Wassersug 2000; Hoff and Wassersug 2000), and tail damage is of less consequence to tadpole survival relative to the lethality of body strikes (Van Buskirk et al. 2003).

In addition to shape, tadpole size can directly influence the outcome of predatory interactions in two, nonexclusive mechanisms. First, large tadpoles may escape predation by exceeding the handling capacity (e.g., gape-limitations) of predators (Cronin and Travis 1986). Growing to a size which exceeds the handling capacity of predators would be most effective against smaller predators, such as invertebrate predators (Cronin and Travis 1986) relative to larger predators, like many predatory fish (Semlitsch and Gibbons 1988). If this form of size-mediated survival is important, we would expect to see larger tadpoles early in development in habitats dominated by invertebrate predators relative to those tadpoles inhabiting ponds with fish predators. Secondly, fast-start performance may scale with body size (Wilson and Franklin 2000; Eidietis 2005; Johnson et al. 2008), thus large tadpoles could be at an advantage, not as a consequence of being difficult for the predator to handle but because they have increased locomotor performance relative to smaller tadpoles. In this scenario, one would expect fast-start and body size to have a positive relationship in habitats with predatory fish.

Predator–prey ecology of morphological variation in anuran larvae has been well documented often by experimental rearing studies (e.g., Van Buskirk et al. 1997, 2003; Relyea 2002; Relyea and Auld 2005; Teplitsky et al. 2005; El Balaa and Blouin-Demers 2013), yet field studies have been less common (Van Buskirk 2009). However, the documentation of natural variation is critical for two reasons. First, documenting natural variation is vital in the development of novel research questions (i.e., informed experimental studies; Van Buskirk 2009). Second, experimental studies provide verification of the conclusions drawn by experimental studies (Van Buskirk 2009). We examined the relationship between tadpole morphology locomotor performance in the Bronze Frog (*Lithobates clamitans*) which can be found in semi-permanent to permanent aquatic habitats (C. K. Adams and D. Saenz unpubl data; Babbitt et al. 2003). Semi-permanent ponds are dominated by invertebrate predators and permanent ponds with fish predator (Wellborn et al. 1996; Babbitt et al. 2003). Previous work (see above) suggests that in the presence of predatory fish, tadpoles should exhibit small bodies and large tail muscles which will increase fast-start locomotor performance. In the presence of larval invertebrate predators, tadpoles should exhibit a medial deep tail fin which is associated with the tail-lure hypothesis. In addition, we evaluate the role these divergent predator regimes have in influencing body size. We expect tadpoles from invertebrate-dominated ponds to develop large size early in development to exceed the handling capacity of these predators. Conversely, large size may also be advantageous in the presence of larger fish predators but not to limit handling ability, but instead as a result of increased fast-start performance and should manifest as a positive relationship between size and fast-start performance.

## Methods

### Specimen collection

Ponds in the Davy Crockett and Angelina National Forests were extensively sampled using dipnets, funnel traps, seines and by hook and line to evaluate the anuran and predator community. We collected *Lithobates clamitans* tadpoles from five fish-dominated and five invertebrate predator-dominated ponds and transported them to the laboratory facilities at the USDA Southern Research Station in Nacogdoches, Texas. Tadpoles were housed in plastic tubs (33 cm × 20.3 cm × 12.7 cm) with aeration, dechlorinated water and fed Tetramin Fish Flakes.

### Swimming performance trails

We used 25 tadpoles from each pond in burst speed swimming performance trials. Trials were only performed with specimens with intact tails and no evidence of previous tail damage. A tadpole was placed individually in an acrylic tank (30.5 cm × 30.5 cm × 5 cm) filled with 3 cm of dechlorinated water and allowed to acclimate for 15 min before the trial began. The tadpole was then startled by probing the lateral ventral portion of the tadpole’s tail. Between trials, the tadpole was allowed 10 min to recover. Trials continued until three trials could be obtained where the tadpole gave significant effort (determined subjectively in comparison with repeated startle events across many individuals) and was fully visible in the video play back. Trials were filmed using a high-speed camera (Photron Fastcam PCI R2; San Diego, CA) recording at 125 frames per second. We digitized the center of the tadpole’s body in each frame by eye. This was performed for the three trials, and the maximum velocity was retained for analysis. Similar methods have been used previously to measure tadpole fast-start performance (Dayton et al. 2005; Johnson et al. 2008). Performance trials were conducted within 24 h of collection. At the conclusion of the trial, each tadpole was euthanized using MS-222 and its developmental stage was determined (Gosner 1960).

### Morphometrics

**Figure 1:**
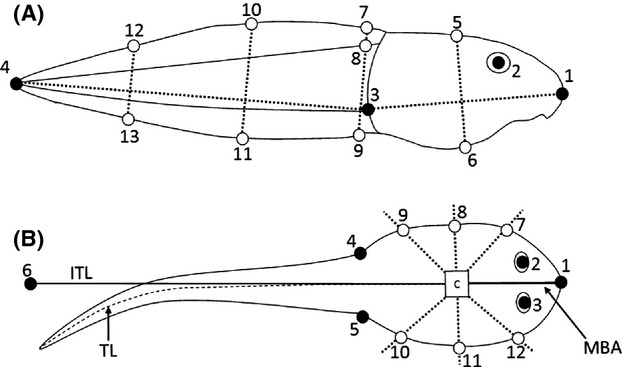
Illustration of the lateral (A) and dorsal (B) digitizing scheme used in this study. For the dorsal digitizing scheme, C = body centroid; MBA = main body axis; TL = tail length; and ITL = inferred tail length. Traditional landmarks are shown as solid points and semi-landmark as open points.

We collected a digital image of the dorsal and lateral view for each specimen using a digital camera (Canon EOS 350D; 105 mm lens) mounted to a copy stand. From each lateral image, coordinates for 13 landmarks were digitized (Fig.1A). Landmarks 1–4 were identified as the tip of the snout (1), the center of the eye (2), ventral insertion of the tail musculature, and the tip of the tail (4; Fig.1A). Landmarks 5–13 were semi-landmarks and were digitized on the body by projecting two chords (dotted lines; Fig.1A) on the body and tail, respectively. This digitizing scheme has been used previously to measure tadpole morphological variation (Dayton et al. 2005; Johnson et al. 2008).

From the dorsal images, 12 landmarks were digitized (Fig.1B). Landmarks 1–6 were identified as the tip of the snout (1), insertions of the tail musculature to the body (5 and 6; Fig.1B). To account for curvature of the tail, we measured the length (mm) from the centroid (“C”; Fig.1B) to the tip of the tail following the midline of the body and tail (“TL”; Fig.1B). This distance was used to infer the straightened tail length (inferred tail length = “ITL”; Fig.1B) and the position of the tip of the tail if the tail was strait, that is, relative to the mid-body axis (“MBA”; Fig.1B). Landmarks 7–12 were semi-landmark and were digitized by radiating at 45° (landmark 7), 90° (landmark 8), 135° (landmark 9), 225° (landmark 10), 270° (landmark 11), and 315° (landmark 12) relative to the MBA (Fig.1B). Symmetric landmarks such as those on the body can inflate the degrees of freedom in shape analysis (Zelditch et al. 2004). Thus, landmarks from one side of the body were reflected back onto the other side (3 to 2, 12 to 7, 11 to 8, 10 to 9, and 5 to 4) and the landmark pairs averaged (Zelditch et al. 2004). These seven landmarks (landmark 1, the five reflected and averaged landmarks and the tip of the tail) were used to estimate shape parameters in our statistical analysis, and the 5 “symmetrized” landmarks were back reflected for visualizations (Zelditch et al. 2004). Thin-plate spline visualizations were used to aid the interpretation of morphometric differences (Zelditch et al. 2004).

Scaled landmark coordinates (mm) were subjected to generalized Procrustes superimposition (i.e., alignment; Bookstein 1991; Zelditch et al. 2004). Semi-landmarks were properly accounted for during landmark alignment (Bookstein 1991; Zelditch et al. 2004). From the scaled, aligned coordinates, we estimated partial warps which describe localized shape variation and two uniform components describing uniform shearing in the X and Y dimensions, respectively (Rohlf et al. 1996; Zelditch et al. 2004). Alignment and estimation of partial warps and uniform components were performed separately for the lateral and dorsal landmark datasets. Lateral morphological data consisted of 20 partial warps and two uniform components and the dorsal dataset included eight partial warps and two uniform components. For each view, we estimated a body size statistic, centroid size, as the square root of the sum squared distances for each individual’s landmark configuration to its centroid (Zelditch et al. 2004). Lateral and dorsal centroid size measures were summed and log-transformed. Alignment, estimation of partial warps and uniform components and centroid size were performed using tpsRelw (Rohlf 2010).

### Statistical analysis

#### Differences in performance between predator regimes

We determined whether fast-start differed between predator regimes using analysis of variance (ANOVA) which included speed as the dependent variable and predator regime (fish or invertebrate) and pond nested within predator regime as independent variables. For this and all subsequent models which include the term pond nested within predator regime, F approximation using Wilk’s Lambda is given.

#### The relationship between morphology, swimming performance, and predator regime

If predator regime is generating divergent selection on morphology, we expect to see differences in morphological variation between tadpoles from invertebrate ponds relative to individuals from fish-dominated ponds. Furthermore, if this hypothesized morphological divergence between predator regimes underlines differences in fast-start performance, we predict that predator morphology of tadpoles will closely match differences in fast-start swimming performance. These two predictions were evaluated using multivariate analysis of covariance (MANCOVA) coupled with canonical variate analysis (CVA). Dependent variables for the MANCOVA model included partial warps and uniform components and independent variables included predator regime, pond nested within predator regime, and log centroid size to account for allometric affects. Two models were performed separately for lateral and dorsal data sets. For both lateral and dorsal data sets, only the first canonical variate (CV 1) axis had an eigenvalue >1 and was thus retained for further analysis. Lateral and dorsal CVA scores were regressed on fast-start speed, respectively.

Performing CVA with geometric morphometric data, while common (Adams et al. 2004) has been criticized as it may result in distorted visualizations thus rendering an incorrect understanding of shape change between groups (see Mitteroecker and Bookstein 2011). Three alternatives, to our knowledge, have been suggested, first, between-group principal component analysis (Mitteroecker and Bookstein 2011), and second, eigen-decomposition of the effect sum-of-squares cross-product matrix (SSCP in SPSS or the H matrix in SAS terminology) from the MANCOVA model (Langerhans 2009b). Both the between-group principal component analysis and the eigen-decomposition of the SSCP matrix methods would render morphological axis which would then be regressed on swimming performance, similar to our CVA approach. The final method involves comparing the angle between vectors of regression coefficients predicting morphological variation between predator regimes and those describing swimming performance, that is, vector correlation (Klingenberg 1996; Zelditch et al. 2004; Collyer and Adams 2013). We performed all three alternative methods and found qualitatively identical results both statistically and visually to the CVA. However, for the sake of simplicity, we present only the CVA results.

To visualize differences in morphology and swimming performance, we used nonparametric thin-plate spline regression to create a performance surface (Arnold 2003; Lee et al. 2008) describing the relationship between the lateral and dorsal CV 1 axes (visualized using TPS regr; Rohlf 2009) and fast-start speed (Fig. 3).

#### Differences in size, development, and fast-start performance

To evaluate the relationship between tadpole size and development (Gosner stage) with respect to predator regime, we performed analysis of covariance (ANCOVA) where log centroid size was dependent on pond (nested within predator regime), predator regime, Gosner stage, and an interaction effect of Gosner stage and predator regime. The ANCOVA approach allows the hierarchal evaluation of two hypotheses: First, do the two predator regimes differ with respect to the relationship between Gosner stage and body size? This question was addressed by evaluating the homogeny of slopes, specifically the interaction term of Gosner stage and predator regime. A significant interaction term is interpreted as evidence that the slopes are different from one another (Zar 1999). If the interaction effect was found to be nonsignificant, the second hypothesis that the elevation (i.e., intercept) of the slope for each predator regime differed from one another could then be evaluated. This was carried out by dropping the interaction term and testing the significance of the predator regime term (Zar 1999). We also applied this same ANCOVA approach to evaluate the relationship between size and fast-start speed where fast-start speed was dependent on pond (nested within predator regime), predator regime, log centroid size, and an interaction effect of log centroid size and predator regime. Statistical analysis was performed in JMP (version 11, SAS, Cary, NC).

## Results

### Fast-start speed and predator regime

**Figure 2:**
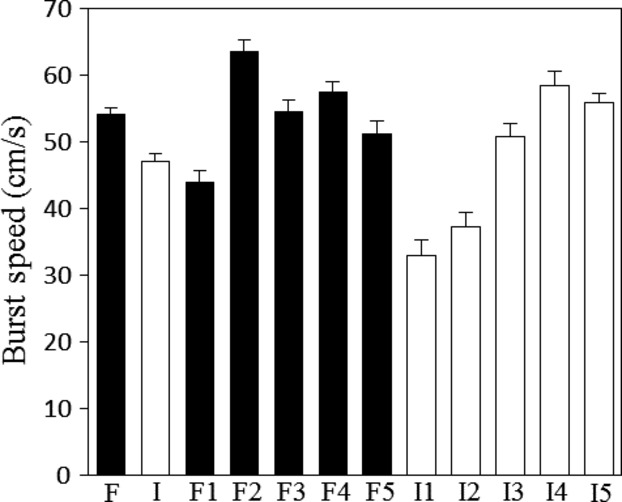
The means and standard errors for fast-start speed for ponds with fish predators (F1–F5) and ponds with invertebrate predators (I1–I5). Global means are shown to the left of each panel in columns F (fish) and I (invertebrate).

Tadpoles from ponds with fish predators were on average faster (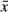 = 54.1 cm/sec, SE = ±0.98) than tadpoles from ponds dominated by invertebrate predators (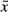 = 47.02, SE = ±1.26; Table1; Fig.2). Significant variation in speed between ponds was also observed (Table1; Fig.2).

**Table 1:** ANOVA results showing differences in fast-start performance by predator regime and pond nested within predator regime (F approximation based on Wilk’s Lambda)

Effect	F	Num DF	Den DF	*P*
Pond (Predator regime)	26.014	8	240	<0.0001
Predator regime	35.568	1	240	<0.0001

#### Morphology, fast-start speed, and predator regime

**Figure 3:**
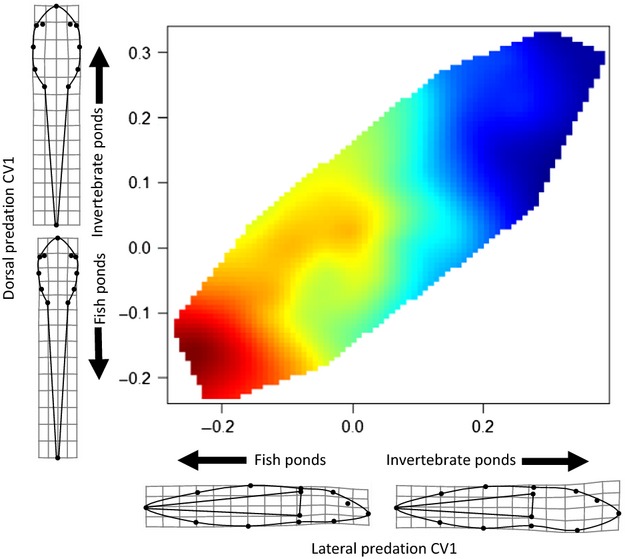
Surface plot showing lateral CV1 and dorsal CV1 in relation to fast-start speed; red denotes faster and blue slower.

Tadpoles with relatively large tail muscles, longer tails, smaller bodies, and posteriorly and ventrally shifted eyes were associated with ponds with fish predators, while tadpoles from invertebrate ponds had larger bodies, shorter tails, relatively smaller tail muscles, and anteriorly and dorsally positioned eyes, as suggested by the predator regime effect in our MANCOVA model (Table2) and visualizations (Fig.3). Multivariate allometric effects were evident as the covariate of log centroid size effect was significant in both lateral and dorsal MANCOVA models (Table2).

**Table 2:** Results for lateral (A) and dorsal (B) MANCOVA models where morphology was dependent on pond nested within predator regime (F approximation based on Wilk’s Lambda), predator regime and log centroid size

Effect	F	Num DF	Den DF	*P*
(A) Lateral				
Pond (Predator regime)	4.714	176	1661.4	<0.0001
Predator regime	13.934	22	218	<0.0001
Log centroid size	9.891	22	218	<0.0001
(B) Dorsal				
Pond (Predator regime)	8.633	80	1467.3	<0.0001
Predator regime	24.967	10	230	<0.0001
Log centroid size	25.012	10	230	<0.0001

The fish predator-associated morphology swam fastest (Fig.3). Both the lateral and dorsal CV 1 axes were significantly related to fast-start speed, as suggested by regression analysis (lateral CV 1 model: *β *= −54.01, SE = 5.649, *t* = −9.56, *P* = <0.001, *r*^2^ = 0.27; dorsal CV 1 model: *β *= −51.338, SE = 6.985, *t* = −7.35, *P* = <0.001, *r*^2^ = 0.18).

#### Differences in size, development, and fast-start performance

**Figure 4:**
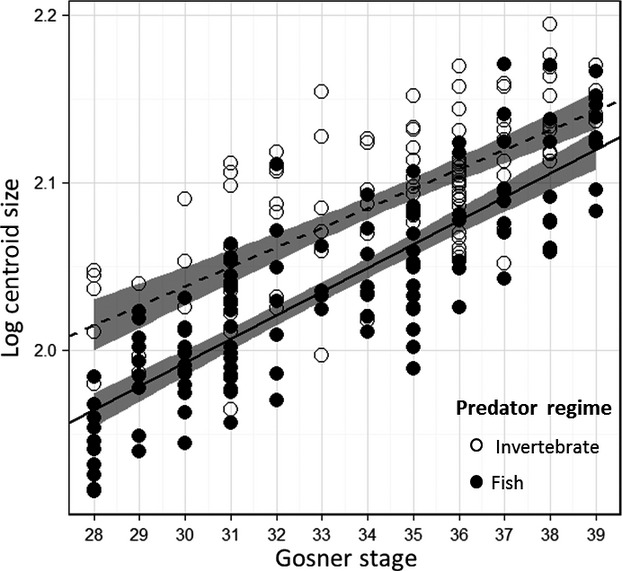
The relationship between Gosner stage and log centroid size for tadpoles from ponds with fish and invertebrate predators.

Early staged tadpoles from invertebrate ponds were, on average, larger than tadpoles from fish-dominated ponds, but this difference in size disappeared later in development (Fig.4) as suggested by the significant interaction term of Gosner stage and predator regime from the ANCOVA model (Table3). Pond (nested within predator regime), predator regime, and Gosner stage were also significant (Table3).

**Table 3:** ANCOVA results where log centroid size was dependent on pond nested within predator regime (F approximation based on Wilk’s Lambda), predator regime, Gosner stage, and the interaction of predator regime and Gosner stage

Effect	F	Num DF	Den DF	*P*
Pond (Predator regime)	20.27	8	226	<0.0001
Predator regime	124.90	1	226	<0.0001
Gosner stage	425.27	1	226	<0.0001
Predator regime ^*^ Gosner stage	4.90	1	226	0.028

The allometric scaling of tadpole size and fast-start speed did not significantly differ between predator regimes (interaction term of size and predator regime, *F*_1,227_ = 0.729, *P* = 0.39). We removed the nonsignificant interaction term to evaluate the overall relationship between size and speed and the elevation of the slopes between predator regimes for both variables. For tadpoles of either predator regime, burst speed scaled positively with size. However, the marginally significant effect of size (*F*_1,228_ = 3.36, *P* = 0.07) suggests that size is at best weakly associated with fast-start swimming performance. The predator regime effect was significantly different (*F*_1,228_ = 34.79, *P* = <0.001) suggesting that while the overall relationship between size and performance is weak, the elevation for each slope differed. The pond effect (nested within predator regime) was also significant (*F*_8,228_ = 3.36, *P* = <0.0001).

## Discussion

Our results suggest that fish and invertebrate predators exert a strong influence on size, shape, and swimming performance of *L. clamitans* tadpoles. However, these differences in morphology and performance may arise from several, nonmutually exclusive evolutionary processes. For example, predation may alter the distribution of traits both within and among generations (Endler 1986; Nosil and Crespi 2006). Furthermore, selection across environmental gradients generates divergent natural selection which may lead to the evolution of locally adaptive, static phenotypic differences between populations (canalization) or adaptive phenotypic plasticity (e.g., inducible defenses) depending on the degree of gene flow relative to generation time (grain size) between environments (Levins 1968; DeWitt and Scheiner 2004; Lind et al. 2011). Thus, organisms which inhabit divergent environments but have high gene flow between those environments are at a selective advantage to display plastic rather than canalized phenotypes (Sultan and Spencer 2002; DeWitt and Scheiner 2004). This is a common scenario for organisms with complex life-history strategies, such as anurans (Benard 2004). In the presence of predators, anuran larvae exhibit inducible morphological traits which increase survival in the presence of predators (Van Buskirk et al. 1997, 2003; Relyea 2002; Kishida and Nishimura 2005; Relyea and Auld 2005; Teplitsky et al. 2005; El Balaa and Blouin-Demers 2013). Alternatively, the ponds sampled in the current study are not particularly close (mean pair-wise distance = 33.4 km, SE = ±4.5 km), which could be a significant obstacle to dispersal and result in local adaptation. It is very likely our results represent all three of these factors, selection within a generation, canalization, and a plastic response to the presence of predators. The strength of these nonmutually exclusive effects can be inferred by rearing tadpoles in the presence of both predator types and a control. However, regardless of the process (selection within a generation, canalization or plasticity), it is likely that the predominate factor defining the observed variation in morphology and performance in *L. clamitans* larvae is divergent natural selection generated by the invertebrate and fish predator environments (see Van Buskirk 2002 for a similar study system).

However, our results cannot exclude the possibility of other selection forces influencing tadpole morphology and swimming performance. For example, leaf litter quality, pond permanence, forest canopy closure, aquatic vegetation, and density of competitors can effect anuran morphology (Van Buskirk 2009; Stoler and Relyea 2013). Van Buskirk and Arioli (2005) found that *Rana temporaria* tadpoles from permanent ponds with an open canopy produced a morphology very similar to the predator-induced morphology (tail-lure morph). While other factors, in addition to predator regime likely play a role in determining morphology and locomotor performance in *L. clamitans*, the question remains whether these latent variables have completely misguided our interpretation that predator regime (particularly the presence of fish predators) is a large factor dictating the observed phenotypic differences. We argue this is unlikely for four reasons. First, the presence and type of predator have previously been defined as a major factor effecting anuran morphological variation (Van Buskirk 2009; Van Buskirk et al. 1997; Van Buskirk 2002; Relyea and Werner 2000; Relyea 2003, 2004; and many more). Second, our findings are similar to previous work demonstrating that many fish predators (particularly those that are active foragers) strongly influence morphology and swimming performance in some anuran tadpoles (Teplitsky et al. 2003, 2005; Wilson et al. 2005; Touchon and Warkentin 2008) and that increased swimming performance may improve survival with active foraging fish predators (Teplitsky et al. 2005). Counter to this argument, Sosa et al. (2009) found that *L. yavapaiensis* reared in the presence of *Lepomis cyanellus* (a fish species common in our system) did develop divergent morphological features such as a larger tail muscles, but these induced features did not increase survival. Third, tadpole morphology in fish ponds meets predictions from first principles; animals with larger propulser will have greater fast-start performance (Webb 1984) and match previous work with anurans (Johnson et al. 2008; Arendt 2010). Finally, a very similar relationship between morphology and swimming performance, that is, a larger propulser facilitates greater fast-start performance, has been documented in prey fish occupying similar fish-dominated predator regimes (Langerhans et al. 2004; Domenici et al. 2008). We discuss these factors in more detail below.

The relationship between morphology and locomotor performance was particularly striking in fish-dominated ponds. We found that tadpoles from fish predator ponds had relatively long tails, large tail muscles, small bodies and swam faster compared to tadpoles from invertebrate-dominated ponds (Figures2 and 3). We suggest that morphological features seen in tadpoles from fish ponds causally influence fast-start speed as these features are congruent with expectations from biomechanics (a smaller anterior region and larger propulsor should increase unsteady swimming performance) and have been documented in other anuran larvae as well as fish as improving fast-start performance (Arendt 2010; Langerhans and Reznick 2010).

The relationship between morphology and fast-start swimming performance seen in ponds with fish is likely advantageous in the presence of these predators. Unfortunately, only one study has, to our knowledge, evaluated tadpole morphology, locomotor performance, and fitness in the presence of fish predators (Teplitsky et al. 2005). Teplitsky et al. (2005) found that when reared with a fish predator (Three-spined Stickleback, *Gasterosteus aculeatus*), *Rana dalmatina* tadpoles grew longer, deeper tails, deeper tail muscles and shallower bodies. These fish predator-induced tadpoles also had improved fast-start performance and survived better in predation trials with the fish predator, but see Sosa et al. (2009) for a similar study with an different outcome. Small prey fish, which have received more attention, show a well-supported causal link between morphology, swimming performance, and evading attacks from fish predators (reviewed by Langerhans and Reznick 2010). These studies suggest that many fish (e.g., poeciliids) which express smaller anterior bodies and large caudal regions have greater fast-start performance and improved survival in the presence of piscivorous fish (reviewed by Langerhans and Reznick 2010). We suggest that the fish predator-associated morphology and improved swimming performance observed in *L. clamitans* larvae likely facilitate survival in the presence of these predators. However, more work should be carried out by examining this hypothesis in detail.

However, tadpole-induced response to fish predators may be more complex. For example, Relyea (2001a,b) found that wood frog (*L. sylvaticus*) tadpoles reared with The Central Mudminnow (*Umbra limi*) grew deeper tail fins, smaller tail muscles and reduced activity rate but suffered greater predation with these predators. The author also found that *L. clamitans* tadpoles (the species in this study) developed smaller tail muscles and shorter tail fins in the presence of *U. limi* and suffered a lower mortality rate compared to other anuran species studied. These results could indicate that other antipredator traits, such as behavior (Skelly 1994), may play a more direct role in escaping predators in this system. Alternatively, the discrepancy between Relyea’s (2001a,b) findings and our results may reflect differences in foraging tactics between *U. limi* and centrarchids. *Umbra limi* is a sit-and-wait predator (Goolish 1991, 1992), while centrarchids, such as *Micropterus salmoides* and *Lepomis cyanellus* (which are common in our study system), frequently active forage (Savino and Stein 1982). Furthermore, Teplitsky et al. (2003, 2004, 2005) found that *Rana dalmatina* tadpoles developed proportionally larger tail muscles when reared in the presence of the active foraging fish, *Gasterosteus aculeatus,* and that this morphology increased fast-start swimming performance and survival with these predators. Thus, active foraging predatory fish may generate selection for increased fast-start performance (Teplitsky et al. 2005).

Many studies have shown that the medially deep tailfins can be induced by rearing tadpoles in the nonlethal (caged) presence of invertebrate predators such as Aeshnid larvae (McCollum and Leimberger 1997; Van Buskirk 2002; Relyea 2004). The larger tailfin functions to attract predator attacks away from the tadpole’s body to the less vital tail (Van Buskirk et al. 2003; Johnson et al. 2008). Contrary to our expectation, we did not find evidence of the tail-lure morphology, a medially deep tail fin in *L. clamitans* larvae from ponds dominated by invertebrate predators. Similarly, Relyea (2001a) found that *L. clamitans* did not develop deeper tail fins when reared in the nonlethal presence of invertebrate predators, including *Anax junius*. The degree to which the tail-lure morph is induced depends upon the anuran species, the specific invertebrate predators, and their density (Relyea 2001a,b, 2003, 2004). Thus, our result may reflect the findings of Relyea (2001a) which suggest that *L. clamitans* does not develop the “lure morph”. Alternatively, the invertebrate ponds sampled in our study were likely variable in predatory species composition and density, although *Anax junius* was common in all ponds (D. Saenz and C.K. Adams per. obser.). We found that morphology was highly variable between ponds even with in the same predator regime (Fig.2; pond effects were always significant) which may reflect subtlety in predator density and composition.

Typically, larger predators have fewer restrictions regarding gape limitation (Werner 1974; Paine 1976). However, Kishida and Nishimura (2005) found that the larval salamander *Hynobius retardatus* is more gape-limited relative to the smaller invertebrate predator, *Aeshna nigroflava*. Fish in our system are considerably larger than invertebrate predators, for example, *Micropterus salmoides* can reach up to 97 cm and *Lepomis cyanellus* 31 cm (Page and Burr 1991). Relyea (2001b), found the common invertebrate predators *Anax* spp*., Belostoma* spp., and *Dytiscus* spp. range in size from 5.2 cm, 2.2 cm, and 3.8 cm, respectively. The invertebrate predators reported in Relyea (2001b) are similar in size to those in *L. clamitans* ponds (D. Saenz pers. obs.). Furthermore, Hoyle and Keast (1987) evaluated prey size and handling time for *M. salmoides* found that bullfrog (*L. catesbeianus*) tadpoles, which are similar sized if not often larger than *L. clamitans* tadpoles (Martof 1956; Werner and McPeek 1994), were among the easiest prey items for these predators to consume. In the current study, tadpoles from fish-dominated ponds were, on average, 5.03 cm total length with a maximum total length of 7.3 cm (D. Saenz unpubl. data). Therefore, if we consider the maximum reported lengths, *M. salmoides* is more than 13 times larger than *L. clamitans*. Thus, it is unlikely that the fish predators in our system, as adults would be gape-limited with respect to consuming *L. clamitans* tadpoles. If size was an effective antipredator strategy to increase handling difficulty against smaller predators, which in our system are invertebrate predators, there should be a selective advantage to obtaining large size early in development when occupying habitats with invertebrate predators. Our results support this expectation. *Lithobates clamitans* tadpoles from invertebrate ponds were larger early in development, relative to tadpoles from fish ponds (Fig.4). In addition, tadpoles from invertebrate ponds expressed large body size (Fig.3). Size has strong effects on the expression of antipredator behaviors as well as competitive ability. Specifically, larger tadpoles increase foraging activity with little consequence of predation from smaller predators (Formanowicz 1986; Semlitsch 1990; Laurila et al. 1997), and larger bodied tadpoles have longer digestive tracts and greater completive ability (Relyea and Auld 2004, 2005). Thus, *L. clamitans* tadpoles may reach a size refuge where most invertebrate predators are of little threat and thus are less constrained morphologically to grow longer digestive tracts to maximize growth and size at metamorphosis, which is a strong determinate of anuran adult fitness (Berven and Gill 1983; Smith 1987; Cabrera-Guzmán et al. 2013). Furthermore, this offers an additional explanation as to why we did not observed the expected tail-lure morphology. Tadpoles that grow to a size refuge may lack the need for the tail-lure defense and are less constrained to keep digestive tracts short (i.e., make the body a smaller target). Alternatively, the differences in tadpole size seen between the predator regimes may reflect other, latent factors. For example, if temperature differences exist between invertebrate and fish-dominated ponds, it could alter algal composition and anuran growth patterns between the pond types (Alvarez and Nicieza 2002; Skelly et al. 2002; Schiesari 2006). Further work is needed to assess the causal mechanisms affecting tadpole size in this system.

We also suggested that large size could be beneficial in the presence of larger fish predators because of larger tadpoles having greater fast-start speed (Wilson and Franklin 2000; Eidietis 2005; Johnson et al. 2008). This hypothesis would be supported by a strong relationship between size and fast-start performance for tadpoles from ponds with fish predators. Our results do not suggest that size scales with fast-start speed in this system. Thus, fast-start speed may be an effective antipredator tactic in ponds with fish predators, it was shape and not size which appears to be a greater determinate of locomotor performance.

In addition to relative proportions of the body, head, and tail, predator regime appears to also drive morphological variation in eye position in *L. clamitans* tadpoles. In ponds with fish predators, tadpole eye position was positioned more posteriorly and ventrally relative to invertebrate-dominated ponds. Variation in light environment (Tobler et al. 2008), foraging ecology (Nyboer and Chapman 2013) and predator regime (Langerhans et al. 2004) have associated with size and position of the eyes in fish, but our study is the first, to our knowledge, to document this pattern in anurans. Similar eye position changes have been observed in prey fish inhabiting sites with large fish predators (Langerhans et al. 2004; Gomes and Monteiro 2008). It has been suggested that the anterior ventral orientation of the eyes may assist with predator avoidance by allowing prey a wider field of view and thus greater reaction time to an attack (Langerhans et al. 2004). In habitats with active foraging predators, such as predatory fish, the early detection of predators would be beneficial to prey. While plausible, this hypothesis is currently untested, but our results do suggest this phenomenon occurs in amphibians.

A considerable number of manipulative studies (rearing, predator trials, etc.) have been conducted to test hypotheses relating to costs/benefits and predator–prey ecology of inducible morphological defenses in anuran larvae (e.g., Van Buskirk et al. 1997, 2003; Relyea 2002; Relyea and Auld 2005; Teplitsky et al. 2005; El Balaa and Blouin-Demers 2013). Field studies have been less common but provide a confirmation of trends described in experimental work and provide material to forge new hypotheses (Van Buskirk 2009). For example, results from experimental studies allowed us to make clear predictions with respect to the patterns of morphological and swimming performance based on the predator communities these anurans occupy. In support of our expectations, we found that *L. clamitans* tadpoles in ponds with predatory fish developed long tails, small bodies, and large tail muscles which likely contributed significantly to the increase in fast-start performance observed in these populations. Conversely, our results do not offer strong support for the occurrence of the tail-lure morphology commonly described in anuran larvae which are reared in the presence of invertebrate predators. Our results do offer an opportunity for size to play a large role in determining survival, yet this idea requires more investigation. In short, we confirm the expectation that morphology and swimming performance of *L. clamitans* larvae in fish and invertebrate predator communities differs dramatically and represents divergent selection imposed by these predator communities.
